# Metadiscourse in helping strategies: a corpus-based analysis of Chinese online psychological counseling responses

**DOI:** 10.3389/fpsyg.2026.1933079

**Published:** 2026-10-28

**Authors:** Yujun Wang, Jingjie Li

**Affiliations:** Institute of Language Sciences, Shanghai International Studies University, Shanghai, China

**Keywords:** counseling strategies, metadiscourse, online counseling, psychological discourse, self-healing, therapeutic alliance

## Abstract

**Introduction:**

Text-based online psychological counseling lacks nonverbal cues, relying primarily on language to sustain the therapeutic alliance and facilitate clients’ self-healing. This communicative constraint makes it important to investigate how counselors use linguistic resources, especially metadiscourse, to achieve these aims.

**Methods:**

To this end, using a corpus-based approach built upon the PsyQA dataset, the present research maps the metadiscourse distributions within six helping strategies in Chinese online counseling responses and further examines how metadiscourse supports exploration, insight, and action.

**Results and discussion:**

This research reveals that each strategy exhibits a characteristic metadiscourse distribution, tailored to its specific discursive function. This consistency suggests that linguistic choices are shaped in a principled manner by the function each strategy is intended to serve. Metadiscourse can therefore be understood as a set of linguistic resources through which counselors fulfill these functions. The implications drawn from these findings are offered as a preliminary reference for applying metadiscourse to counselor training and for informing the design of AI counseling systems aimed at generating contextually appropriate responses.

## 1 Introduction

Psychological counseling is a helping process where counselors foster clients’ self-healing (Hill, 2020). This collaborative endeavor is built upon the foundation of a robust therapeutic alliance (Wampold and Flückiger, 2023). In traditional in-person settings, it is cultivated through an interweaving of verbal and nonverbal cues (e.g., eye contact, facial expressions, and postural resonance) (Pfender and Caplan, 2023; Atzil-Slonim et al., 2023; Rosyidi et al., 2024). In particular, nonverbal cues play a crucial role in facilitating the therapeutic alliance (Gregorini et al., 2025; Lucherini Angeletti et al., 2025). Propelled by the proliferation of digital technology, online platforms have emerged as primary sites for what Tremain et al. (2020) term the “Digital Therapeutic Alliance” (DTA). Online psychological counseling is increasingly favored for its accessibility, scalability, effectiveness, and privacy and trust benefits (Huang et al., 2022; Li et al., 2023; Ni et al., 2024). Yet, in text-only online environments, the absence of nonverbal cues often results in a physical and affective mismatch (Ramachandran et al., 2023; Aviram and Nadan, 2024). Stripped of nonverbal cues, counselors rely on a sophisticated discursive construction to offset this deficit. Consequently, linguistic resources are pivotal for bridging the distance inherent in “text-based communication” (Antoniou et al., 2022, p. 18), building the DTA, and ultimately fostering the clients’ self-healing.

Research indicates that strategic language use is critical to addressing the challenges of missing nonverbal cues in online counseling (Rosyidi et al., 2024; Huang et al., 2025), highlighting the need for therapist adaptability in digital settings (Smith and Gillon, 2021). Such linguistic compensation is evidenced by the adequate establishment of therapeutic alliances in online counseling (Cook and Doyle, 2002). Prominent yet frequently overlooked among linguistic resources lies metadiscourse, conceptualized as a “recipient design filter” (Hyland and Jiang, 2024, p. 412). It illustrates “how communication is shaped to appeal to the current interactants” (Hyland et al., 2022, p. 1). In this sense, its value lies in “how something is said” rather than “what is said”. Such a focus resonates with the “purposeful and strategic” (Zheng et al., 2025, p. 2) feature of counselors’ language.

While the use of metadiscourse is well-substantiated in health communication (e.g., Ryu et al., 2023; Zhang, 2025), its application in the affect-intensive and relationship-oriented context of psychological counseling has received limited attention. Meanwhile, although psychological research has identified counseling strategies (e.g., Shah et al., 2022; Miner et al., 2022; Li M. et al., 2025; Zheng et al., 2025), the underlying metadiscourse distribution remains largely underexplored. Notably, Hill’s (2009, 2020) helping strategies (including approval and reassurance, restatement, self-disclosure, etc.) are designed to build the therapeutic alliance and mobilize the clients’ self-healing capacities. However, whether each strategy possesses a characteristic metadiscourse distribution remains unclear. In this research, self-healing follows the conceptualization of clients as active agents who draw on their capacities to change (Bohart and Tallman, 1999; Hill, 2020). Hill (2020) proposes that counselors facilitate clients’ self-healing by guiding them through exploration, insight, and action. Following this approach, this research analyzes metadiscourse distributions within strategies to support this process. To address the gaps identified above, this research seeks to map the metadiscourse distributions by analyzing the PsyQA dataset (Sun et al., 2021) of authentic Chinese online counseling responses annotated via Hill’s (2009) helping skills. Our findings aim to provide fine-grained evidence of how metadiscourse helps to fulfill counseling strategies in service of clients’ self-healing.

## 2 Literature review

### 2.1 Metadiscourse

At the conceptual level, metadiscourse is fundamentally defined as “discourse about discourse or communication about communication” (Vande Kopple, 1985, p. 83). More specifically, it refers to linguistic resources that speakers employ not to add propositional content, but to convey their own stance, draw the audience into the exchange, and shape how the text itself is organized (Hyland, 2005). Positioned as “a coherent set of interpersonal resources” (Hyland and Jiang, 2024, p. 414), it constitutes the relational scaffolding necessary to guide recipients through communication. Accordingly, metadiscourse enables speakers to tailor their talk for recipients, “based on an estimation of how best they can be helped to process, comprehend and agree with what is being discussed” (Hyland and Jiang, 2024, p. 412).

Within Hyland’s (2005) interpersonal model, metadiscourse encompasses two types: Interactive resources facilitate discourse structuring and information flow; interactional resources highlight the speaker’s stance and the co-construction of meaning with the recipient. In the context of socially situated interactions, these resources embody speakers’ strategic communicative effort to tailor language to manage the recipients’ interpretations and affective reactions. Consequently, adopting an interpersonal lens on metadiscourse is particularly appropriate for analyzing psychological counseling as a collaborative “social engagement” (Hyland, 2005, p. 4). This perspective aligns with its intersubjective nature as a “talking cure” (Berman, 1985), an inherently interpersonal helping process.

At the applied level, metadiscourse analysis has been extended to diverse registers (Hyland, 2017; Hyland et al., 2022). Although Hyland’s (2005) model was originally developed for academic written discourse, its key premise, that language is shaped through the communicator’s negotiation of interpersonal meanings with an anticipated audience, is not restricted to a monologic or written mode. This premise underlies the model’s documented extension to spoken registers, including political speeches (e.g., Farahani and Kazemian, 2021) and classroom discourse (e.g., Li and Hu, 2026), as well as to health communication, where metadiscourse resources such as hedges, self-mentions, and engagement markers have been shown to negotiate delicate interpersonal interactions.

For hedges, their use is instrumental in navigating the interpersonal dynamics of therapeutic and clinical communication. Working within the broader pragmatic framework of mitigation, Cheng et al. (2023) examined a corpus of real-world therapist-client conversations recorded at two top-tier hospitals in Beijing and found that hedging, among other mitigating strategies, helps participants stay aligned with the goals of the exchange, protect their social rights, and avoid loss of face. These functions are interwoven and collectively contribute to building therapeutic rapport while reducing the likelihood of interpersonal friction. Focusing on depression consultations, Chen and Guo (2024) revealed a more nuanced picture. Their study found that hedges are commonly used by both doctors and patients to perform positive functions like building compliance and showing politeness. Crucially, their research identified practical strategies to mitigate negative effects caused by misused hedges, including self-correction, clarification by others, and linguistic techniques such as rephrasing and carefully judged follow-up questioning (Chen and Guo, 2024). Thus, the aforementioned studies collectively demonstrate that hedges can build rapport and preserve face, yet their effectiveness hinges on precise and context-sensitive deployment to avoid the misunderstandings and distrust that misuse can cause.

For self-mentions and engagement markers, the strategic deployment of personal pronouns serves varied and revealing functions across different health communication contexts. In psychotherapy, Ryu et al. (2023) demonstrated that a high density of first-person singular pronouns in a therapist’s speech correlates with a weaker therapeutic alliance, which they attribute to diminished trusting behavior toward the therapist. In a parallel vein, research on health communication short videos has explored the strategic use of personal pronouns from a multimodal perspective. Zhang (2025) identified the use of personal pronouns as a discursive strategy to foster a sense of closeness and personal relevance. As self-mentions and engagement markers, these pronouns transform one-way knowledge dissemination into interactional communication. However, Zhang (2025) also critically noted that these pronouns, when embedded within a commercialized framework, can potentially amplify perceived health anxieties and stimulate consumer behavior. The contrast between the two studies underscores the context-dependent complexity of self-mentions and engagement markers: their function and impact are fundamentally shaped by the speaker’s role (e.g., therapists versus health communicators), the primary communicative objective (e.g., building a therapeutic alliance versus disseminating knowledge), and the specific medium and genre of interaction.

Despite the established relevance of metadiscourse in interpersonal interactions and its demonstrated applications in general health communication, its specific use within the subfield of online psychological counseling remains promising yet underexplored. In other words, whether the functional profiles derived from broad health communication contexts can be directly transposed to the affect-intensive and relationship-oriented environment of online psychological counseling has yet to be established. Furthermore, existing research has predominantly examined individual metadiscourse markers, overlooking their overall distribution. Therefore, there is a critical need to move beyond examining isolated markers in general health communication, and to decode the holistic metadiscourse distribution in online counseling discourse.

### 2.2 Linguistic research in online psychological counseling

Despite the acknowledged importance of language in online counseling, the current linguistic research in this domain remains fragmented. Existing studies generally fall into two strands:

1. The identification of macro-level counseling strategies. Recent efforts have focused on leveraging Large Language Models (LLMs) to build and evaluate AI counseling systems. These approaches include the following: frameworks that use LLMs to plan and execute high-level support strategy chains for generating psychological support replies (Chen et al., 2025); systems designed with dual-role interaction mechanisms (e.g., therapist and friend) to dynamically adapt between professional guidance and emotional support (Cheng et al., 2026); models that perform sentiment analysis on counseling texts to gauge clients’ emotional tone (Wang et al., 2024); research that classifies therapeutic techniques and assesses clinical content from large-scale counseling messages (Imel et al., 2024); and research that extracts behavioral activation language markers from therapy sessions to inform automated assessment and clinical decision support (Burkhardt et al., 2021). These computational models provide powerful tools for counseling strategy formulation, yet they primarily focus on the macro level. What remains largely unexplained are the precise micro-linguistic configurations that underlie these high-level strategies as they naturally unfold in counseling dialogues.
2. The analysis of linguistic features and their correlations with psychological states and interactional outcomes. For instance, Zheng and Huang (2016) conducted a corpus-based analysis of a Hong Kong online counseling platform. Combining quantitative association tests with qualitative discourse analysis, they examined the content, function, and interactivity of metaphors. Their findings revealed significant associations among the topics discussed, the source domains chosen for metaphors, and the roles of the clients and experts, highlighting metaphors’ strategic use for explanation, advice, and description. Similarly, shifting the focus to peer social support, Li M. et al. (2025) grounded their analysis in signaling theory and identified several linguistic predictors of cognitive change in help-seekers within online communities. Their empirical analysis revealed that supportive comments are more effective when they are less intimate, more emotionally positive, and less specific. Additionally, a higher frequency of first-person expressions and future or present tense words, as well as a lower use of function words, was found to be a significant contributing factor. Yet, while adept at establishing critical correlations between linguistic markers and interactional functions, these studies often treat such markers as isolated resources. Moreover, little is known about the metadiscourse embedded in counseling strategies. Consequently, this approach does not adequately reveal how these markers are coordinated within the unfolding discourse to accomplish specific counseling goals.

To address these limitations, this research adopts a corpus-based quantitative frequency analysis supplemented by qualitative discourse interpretation. Specifically, it aims to answer the following two Research Questions (RQs):

*RQ1*: What types of metadiscourse characterize each of the six counseling strategies?

*RQ2*: How does metadiscourse function within each counseling strategy to support exploration, insight, and action?

## 3 Materials and methods

### 3.1 Data sources

The corpus adopted in this research is derived from the PsyQA dataset (Sun et al., 2021), selected for its authenticity, theory-grounded annotation scheme, and broad academic adoption. First, the dataset guarantees data authenticity by containing large-scale spontaneous counselor responses collected from a leading Chinese online psychological counseling platform. Second, its strategy annotation scheme, rooted in Hill’s (2009) Helping Skills System, enabled manual annotation of 4,012 responses with strategy labels, yielding high reliability (Sun et al., 2021, pp. 1492–1493). The present research draws on this human-annotated subset, whose theory-grounded labels constitute a robust foundation for subsequent metadiscourse analysis. Finally, PsyQA has been widely employed or drawn upon across natural language processing, psychological, and linguistic research, including single-to-multi-turn dialogue expansion (Qiu et al., 2024), strategy-driven response generation (Chen et al., 2025), and the construction of theoretically grounded multi-turn corpora (Du et al., 2026). The extensive and varied applications underscore its enduring validity as a robust, standardized resource bridging computational and human sciences.

In terms of data format, it should be noted that PsyQA responses represent single, asynchronous text-based exchanges between a counselor and a client, rather than ongoing, multi-session psychotherapy; accordingly, the present findings characterize the metadiscourse resources counselors draw on within this specific counseling context instead of psychotherapy in a clinical sense.

In terms of annotation structure, each response in the PsyQA dataset was segmented into consecutive, non-overlapping spans via character indices, with each span assigned a single strategy label; sentences not matching any strategy definition were labeled as “Others” (Sun et al., 2021, p. 1492). Because a single response typically contained multiple spans with different strategy labels (Sun et al., 2021, p. 1493), responses could not be treated as mutually exclusive units for cross-strategy comparison. Metadiscourse markers were therefore counted within each span and aggregated by strategy label for all subsequent frequency and inferential analyses.

### 3.2 Analytical framework

To investigate how counseling strategies are realized through metadiscourse and how these resources contribute to clients’ self-healing, this research proposes an integrated analytical framework. The framework bridges counseling strategies and metadiscourse by synthesizing two well-established theoretical layers: (a) Hill’s (2009, 2020) Helping Skills System, which provides both the theoretical basis for the counseling strategy annotations in the PsyQA dataset (Sun et al., 2021) and the framework of exploration, insight, and action adopted in this research; and (b) Hyland’s (2005) Interpersonal Model of Metadiscourse, applied to map the metadiscourse distributions within each strategy.

The first layer adopts the strategy annotations in the PsyQA dataset (Sun et al., 2021), which are grounded in Hill’s (2009) Helping Skills System. This annotation scheme organizes counseling strategies into six categories: approval and reassurance, restatement, interpretation, information, direct guidance, and self-disclosure. In Hill’s (2020) framework, these strategies are associated with exploration (e.g., approval and reassurance, and restatement), insight (e.g., interpretation), and action (e.g., information and direct guidance). Self-disclosure differs from the other strategies in that its subtypes are linked to different helping intentions: disclosure of feelings or similarities is intended to support exploration; disclosure of insight is intended to facilitate insight; and disclosure of strategies is intended to promote action. Operational definitions and examples are detailed in Tables 1–4.

**Table 1:** Counseling strategies for fostering exploration: definitions and examples.

Strategies	Operational definitions (Hill, 2009; Sun et al., 2021, p. 1492)	Examples (with English translation)
Approval and reassurance	Emotional support, reassurance, encouragement and reinforcement.	非常理解你的困扰, 抱抱你~ Really understand what you are going through. Hug you~
Restatement	A simple repeating or rephrasing of the content or meaning of the question, usually in a more concrete and clear way.	看到您的困惑, 您说想要走出自己内心中的痛苦。 I can sense your confusion, and you are telling me you want to move past this inner pain.

**Table 2:** Counseling strategy for fostering insight: definition and example.

Strategy	Operational definitions (Hill, 2009; Sun et al., 2021, p. 1492)	Examples (with English translation)
Interpretation	Go beyond what the help-seeker has overtly stated or recognized and give a new meaning, reason or explanation.	您的状态比较像是内在缺乏重心和锚定点. It appears that you are experiencing a lack of inner balance, as if you have lost your anchor point.

**Table 3:** Counseling strategies for fostering action: definitions and examples.

Strategies	Operational definitions (Hill, 2009; Sun et al., 2021, p. 1492)	Examples (with English translation)
Information	Supply information in the form of data, facts, opinions and resources.	焦虑情绪很容易引起生理上的不适, 影响身体健康, 也会严重影响睡眠质量. Anxiety can easily cause physical discomfort, affect overall health, and severely disrupt sleep quality.
Direct guidance	Provide suggestions, directives, instructions, or advice about what the help-seeker should do to change.	以上五个问题, 希望您可以静下心来和自己对对话, 看看问题背后我们真正在乎的是什么? For the five questions above, try to quiet your mind and reflect: What truly matters to us beneath these concerns?

**Table 4:** Counseling strategy for fostering exploration, insight, and action: definition and example.

Strategy	Operational definitions (Hill, 2009; Sun et al., 2021, p. 1492)	Examples (with English translation)
Self-disclosure	Reveal something personal about the helper’s non-immediate experiences or feelings.	你所遇到的问题我同样也遇到过. I’ve been through what you are going through.

The second layer examines metadiscourse distributions within counseling strategies. This research draws on Hyland’s (2005) Interpersonal Model of Metadiscourse as its theoretical basis. Interactive metadiscourse (i.e., transitions, frame markers, endophoric markers, evidentials, and code glosses) organizes discourse to guide the clients through a coherent narrative. Complementing this, interactional metadiscourse (i.e., hedges, boosters, attitude markers, engagement markers, and self-mentions) allows counselors to express stance, adjust involvement, and build interpersonal rapport. The contextualized metadiscourse framework, adapted from Hyland (2005, p. 49), is presented in Tables 5, 6.

**Table 5:** Interactive metadiscourse categories (adapted from Hyland, 2005, p. 49).

Categories	Descriptions	Examples (with English translation)
Transitions	Express relations between main clauses	*除此之外*, 你对自己也有观察. *On top of that*, you also have observations about yourself.
Frame markers	Refer to discourse acts, sequences, or stages	*二是*, 我们人类都是逃避痛苦, 和追求愉悦的. *Second*, it is our human nature to avoid pain and seek pleasure.
Endophoric markers	Refer to information in other parts of the text	我觉得首先您可以看看到底是我 *上述提到的* 哪方面的问题. I think you might first consider which of the issues I *mentioned above* applies to you.
Evidentials	Refer to information from other texts	卓别林曾*说过*: “人生近看是悲剧, 远看是戏剧”. *According to* Charlie Chaplin, “Life is a tragedy when seen in close-up, but a comedy in long-shot.”
Code glosses	Elaborate propositional meanings	*也就是说*, 他给予你的, 有部分是满足了你内心需要的. *In other words*, part of what he gave you met your inner needs.

**Table 6:** Interactional metadiscourse categories (adapted from Hyland, 2005, p. 49).

Categories	Descriptions	Examples (with English translation)
Hedges	Withhold commitment and open dialogue	你自己 *似乎* 并没有接纳, 对吗? It *seems* you have not accepted that, right?
Boosters	Emphasize certainty or close dialogue	*当然*, 这不是伤害你的理由. *Definitely*, this is not a reason to hurt you.
Attitude markers	Express counselor’s attitude to proposition	我非常*理解* 你这种行为. I fully *understand* why you did that.
Engagement markers	Explicitly build relationship with the client	*你* 值得爱, 值得尊重, 友谊, 赞美和认同. *You* are worthy of love, respect, friendship, praise, and acceptance.
Self-mentions	Explicit reference to the counselor	*我* 有同感. *I* feel the same way.

### 3.3 Procedures

Annotation followed a theory-driven procedure based on Hyland’s (2005) Interpersonal Model of Metadiscourse. Manual annotation was adopted because metadiscursive features are inherently context-dependent and frequently lack a direct, predictable mapping onto fixed lexical forms (Yu et al., 2024), an approach widely applied in corpus studies of metadiscourse (e.g., Li and Xu, 2020; Chen and Li, 2023; Güçlü and Önem, 2025; Alzahrani and Alharbi, 2026). Within each strategy-labeled span, the smallest contiguous linguistic unit that could potentially serve a metadiscursive function was considered a candidate for annotation. For each candidate unit, its context was examined to determine whether it functioned as metadiscourse, since the same lexical form could carry a metadiscursive function in one context and convey propositional content in another. Adjacent spans within the same response were consulted when necessary. Once confirmed, each metadiscourse instance was assigned to one of the framework’s ten categories.

Prior to formal annotation, the two coders jointly coded a small calibration sample to align their understanding of the coding categories. The two coders then independently annotated the full corpus of 4,012 responses, yielding an inter-coder reliability of Cohen’s *κ* = 0.82. Discrepancies arose in two types of borderline cases: (a) the boundary between propositional content and metadiscursive function, where a lexical item could be interpreted as variously serving a discourse function or conveying content (e.g., “可能” [possibly] functioning variously as a hedge or a proposition-internal modal indicating objective likelihood, or “可以” [could/can] functioning variously as a hedge or a proposition-internal ability or permission modal); and (b) boundaries between metadiscourse categories themselves, where the same form realized different functions across contexts (e.g., “我们” [we] as an inclusive-we engagement marker versus an exclusive-we self-mention). To address these, the coding rules were refined, giving precedence to a function-over-form principle: a lexical item was annotated as metadiscourse only when it served an explicit discoursal or interpersonal function rather than conveying propositional content. All disagreements were resolved through joint discussion and adjudication under the refined rules. As a final step, the two coders cross-checked the entire corpus to confirm that labeling had been aligned with the established rules.

Following annotation, we examined the size distribution of the annotated corpus and determined the normalization strategy for all subsequent frequency analyses. The strategy-annotated corpus varied substantially in size across strategies, ranging from approximately 68,000 characters (Approval and Reassurance) to nearly 1.2 million characters (Interpretation). Because raw frequency counts would conflate differences in metadiscourse usage with differences in corpus size, all frequencies were normalized per 10,000 characters. As an additional check, for each response and strategy, we summed metadiscourse counts and annotated text length across all spans of that strategy within the response and fitted negative binomial regression models predicting these counts from strategy and log-transformed length, for all ten metadiscourse categories. Strategy remained a significant predictor of category counts after controlling for length in all ten categories (likelihood-ratio tests, all ps < 0.001), indicating that the cross-strategy differences are not attributable to differences in text length across strategy types. Finally, to test whether the distribution of metadiscourse categories differed across the six counseling strategies overall, we conducted an omnibus chi-square test of independence on the strategy-by-category frequency data. The distribution of metadiscourse categories differed significantly across strategies (*χ*^2^ = 29,908.53, *df* = 45, *p* < 0.001; Cramér’s V = 0.20).

## 4 Results and discussion

This section addresses the two research questions in turn. For RQ1, it first provides an overview of how the ten metadiscourse categories are distributed across the six counseling strategies (Figure 1), establishing the empirical basis for the strategy-specific analyses that follow. Each strategy’s metadiscourse distribution is then visualized in a radar chart (Figures 2–7), with all charts displayed on a common square-root scale to permit direct visual comparison. To illustrate these patterns, we examined concordances of each strategy’s most frequent metadiscourse categories and selected examples that clearly show how these categories function in context. For RQ2, the observed patterns are interpreted in relation to Hill’s (2020) conceptualization of exploration, insight, and action.

**Figure 1:**
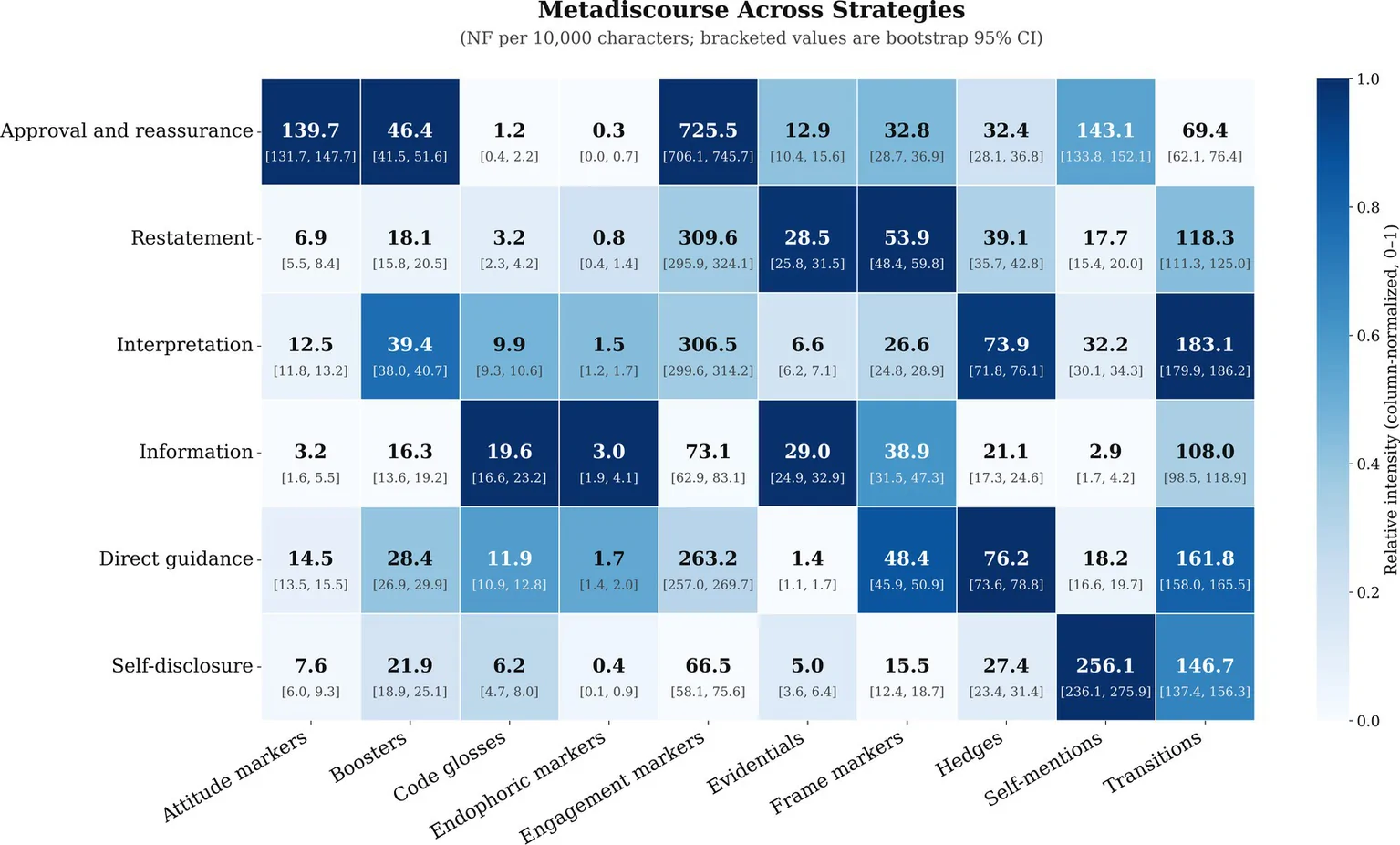
Heatmap overview of metadiscourse distribution across counseling strategies. Colors represent relative intensity within each metadiscourse category (column-normalized, 0–1) and are comparable only within each column. Exact normalized frequencies (NF per 10,000 characters) are reported in each cell. Bracketed values are 95% confidence intervals from a response-level bootstrap procedure (2,000 resamples).

**Figure 2:**
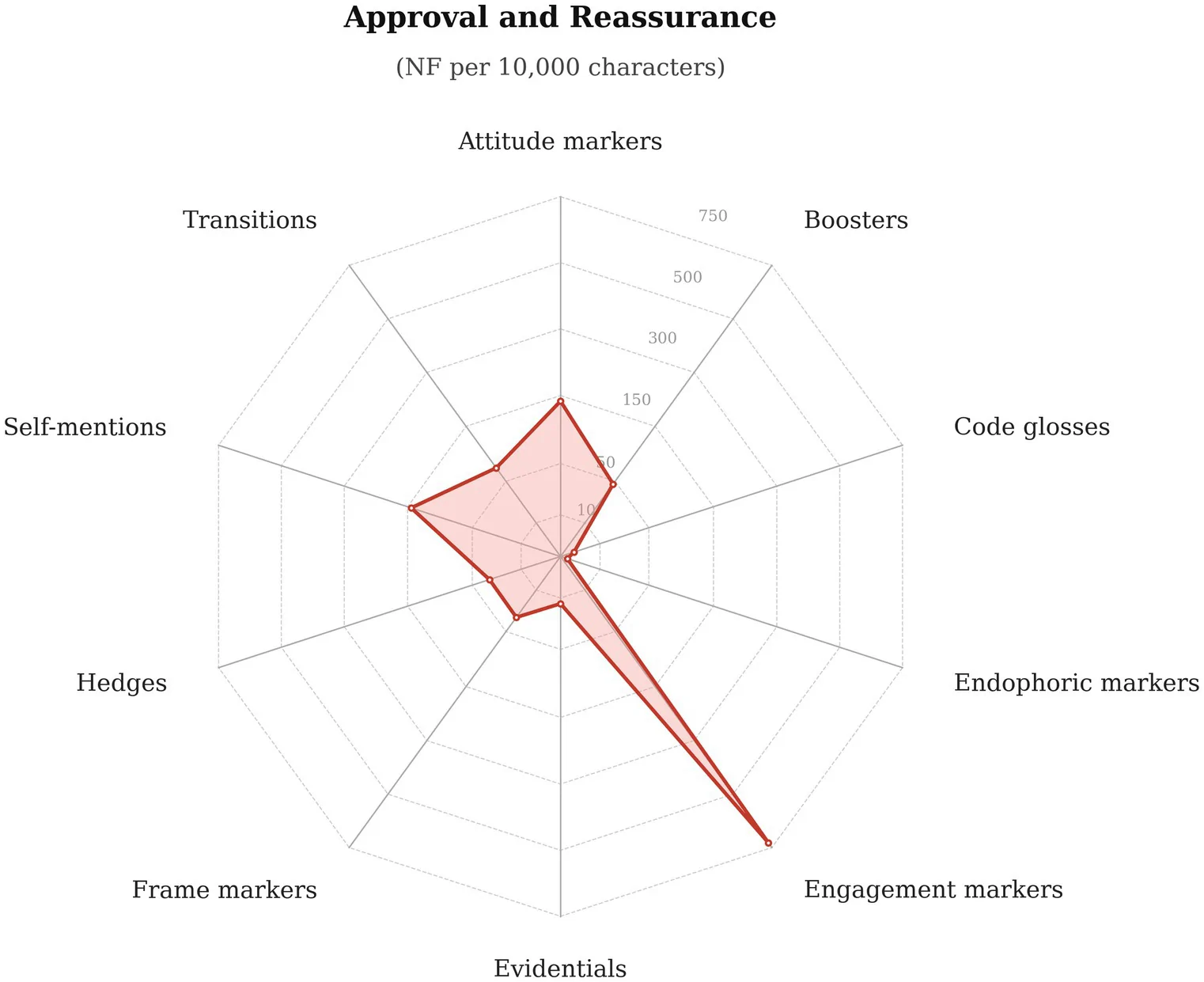
Distribution of metadiscourse in the approval and reassurance strategy. To accommodate the wide range of normalized frequencies across metadiscourse categories, the radial axis uses a square-root scale. The labeled values indicate original normalized frequencies, plotted at square-root-transformed positions. All six radar charts (Figures 2–7) share an identical set of gridlines to enable direct visual comparison across strategies.

**Figure 3:**
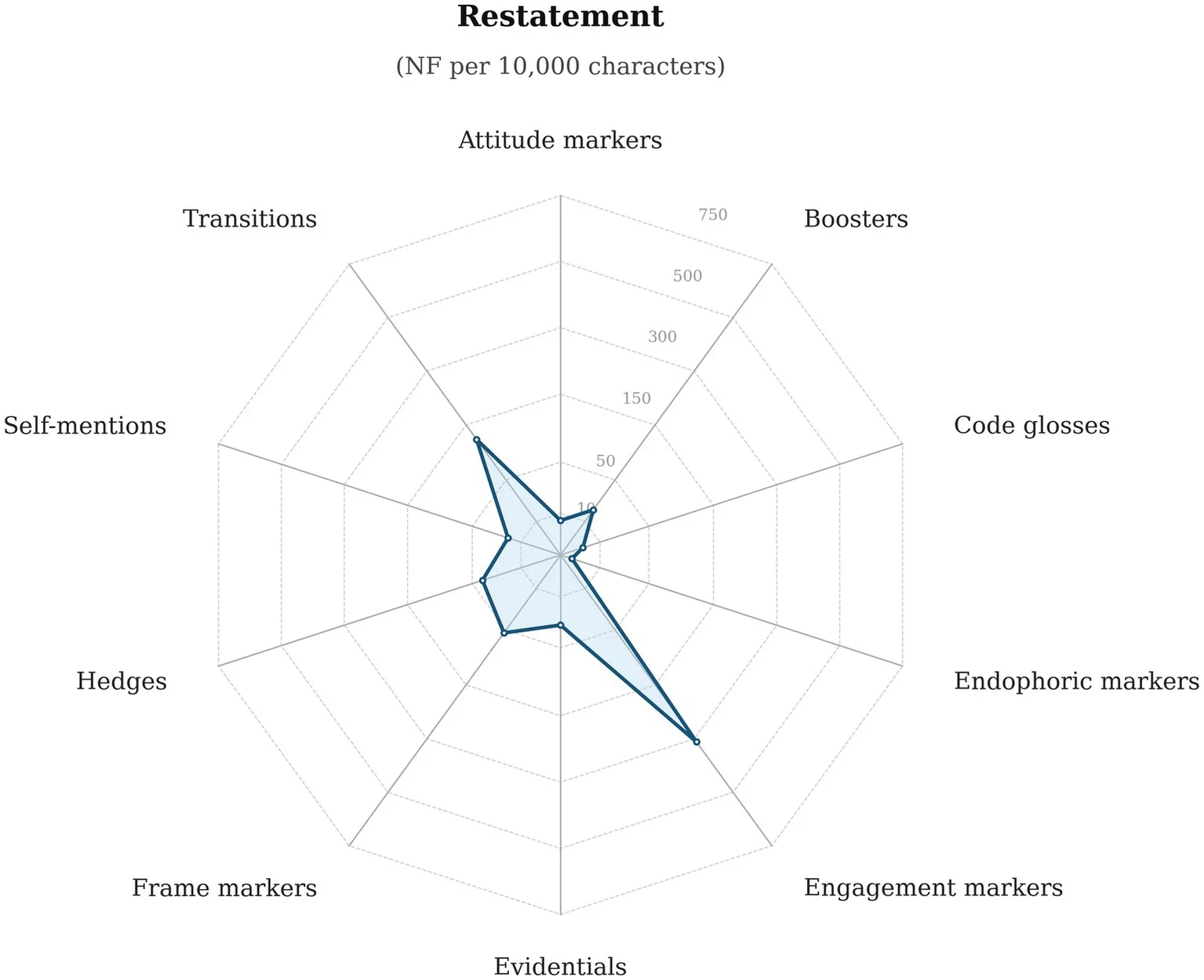
Distribution of metadiscourse in the restatement strategy.

**Figure 4:**
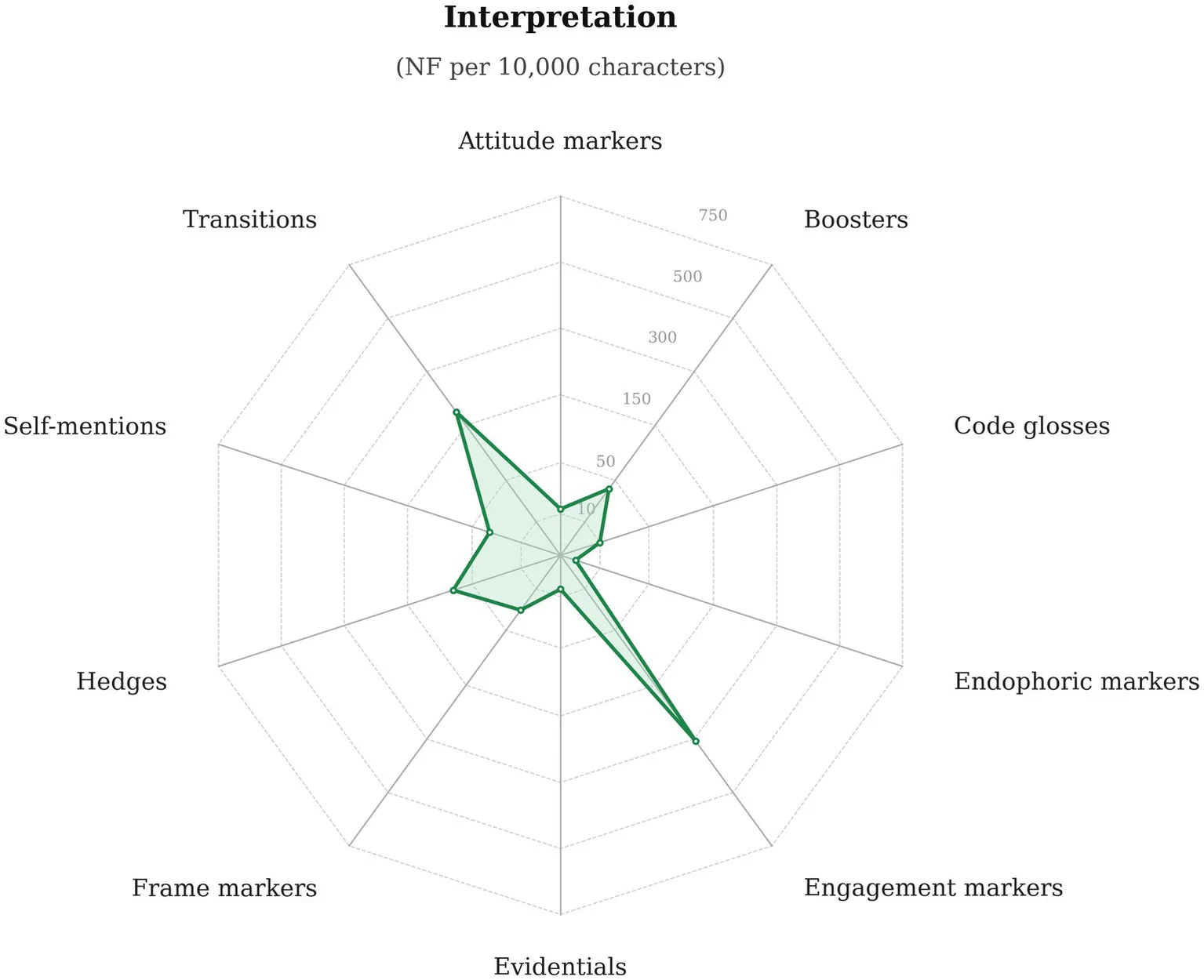
Distribution of metadiscourse in the interpretation strategy.

**Figure 5:**
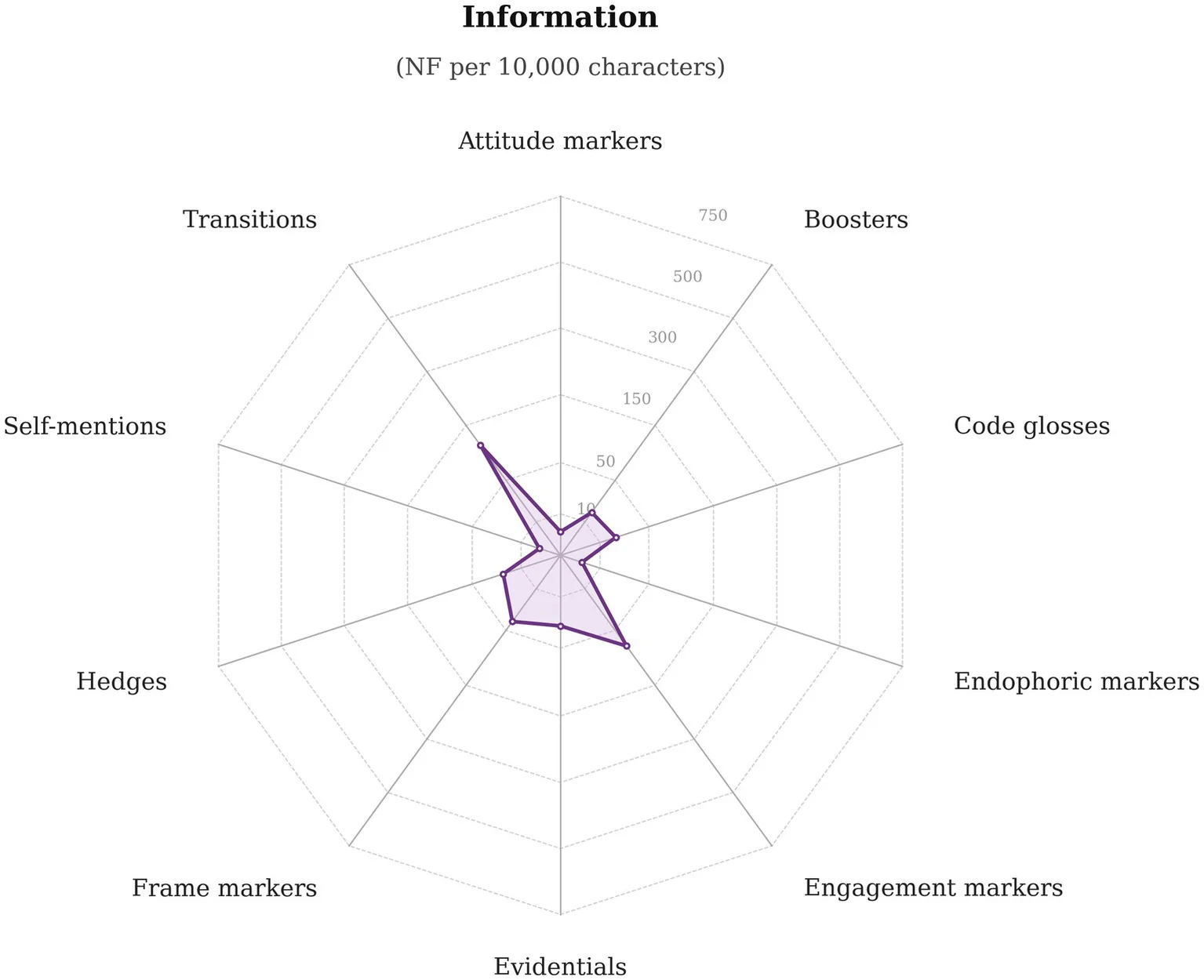
Distribution of metadiscourse in the information strategy.

**Figure 6:**
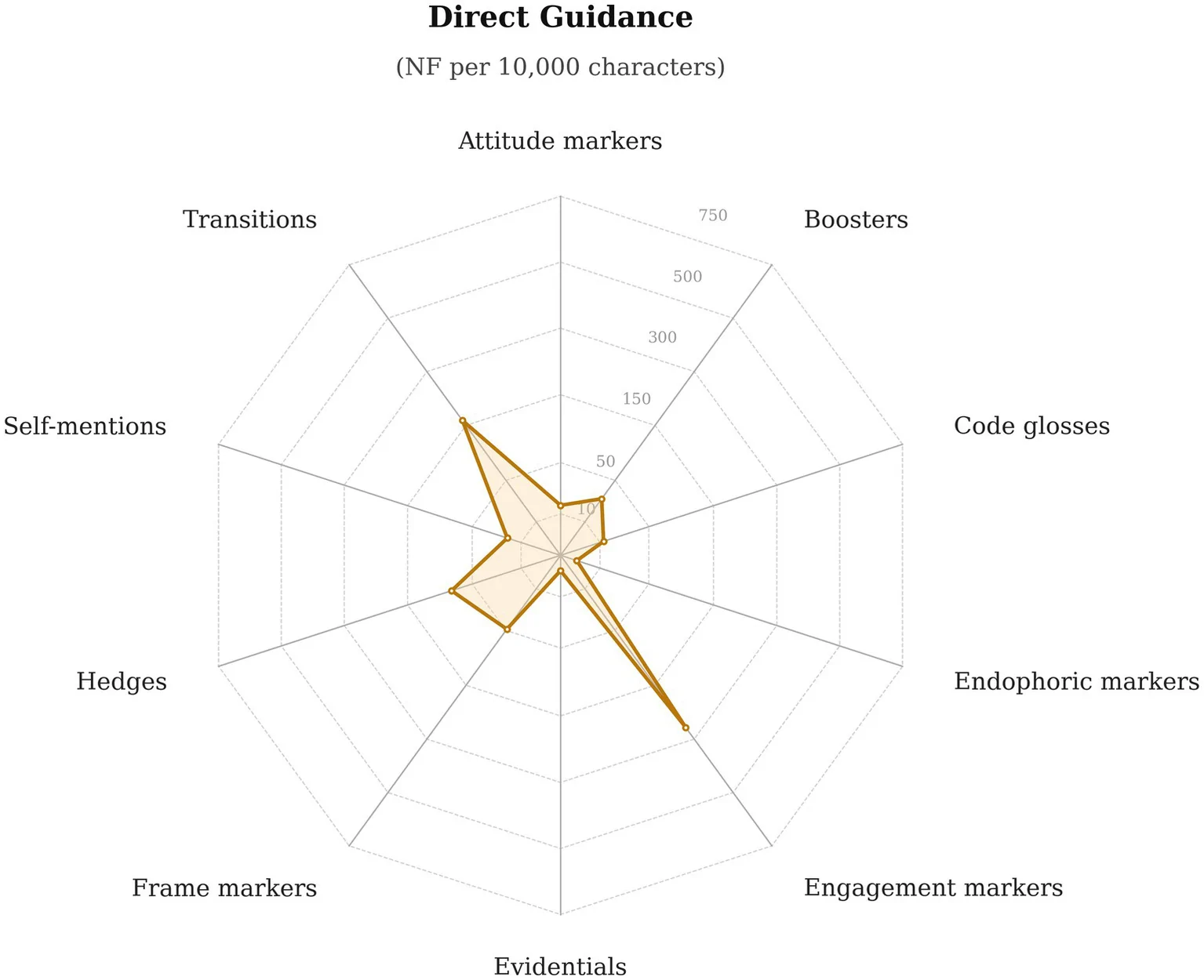
Distribution of metadiscourse in the direct guidance strategy.

**Figure 7:**
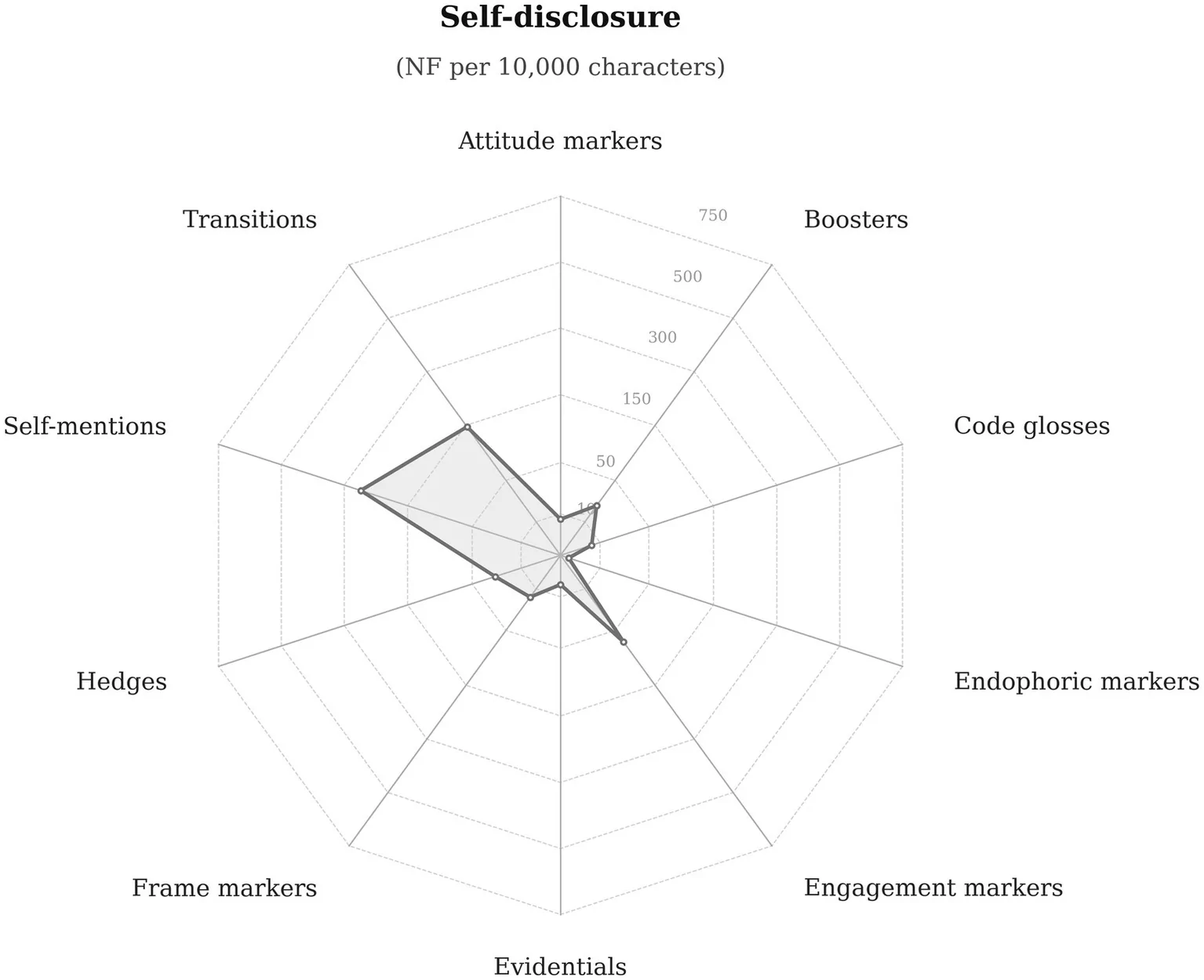
Distribution of metadiscourse in the self-disclosure strategy.

### 4.1 Overview of metadiscourse distribution across strategies

Figure 1 presents the normalized frequency (NF) of each of the ten metadiscourse categories across the six counseling strategies. Three cross-strategy patterns are particularly salient, each examined in greater depth in the strategy-specific analyses that follow.

First, engagement markers are the dominant category overall (with a corpus-wide NF of 285.4), and they are particularly frequent in the two exploration strategies, approval and reassurance (NF = 725.5) and restatement (NF = 309.6). This pattern is taken up in section 4.2.

Second, transitions and hedges, although less frequent overall than engagement markers, are concentrated in interpretation (NF = 183.1 and 73.9, respectively) and direct guidance (NF = 161.8 and 76.2, respectively). This pattern is elaborated in sections 4.3 and 4.4.

Finally, self-mentions occur far more often in self-disclosure (NF = 256.1) than in any other strategy. Within self-disclosure itself, self-mentions occur over three and a half times as often as engagement markers (NF = 66.5), making self-reference the feature that most clearly distinguishes this strategy from the other five. This pattern is discussed further in section 4.5.

Overall, these patterns indicate that the ten metadiscourse categories are not evenly distributed across strategies, motivating the strategy-specific radar profiles examined in closer detail in sections 4.2 through 4.5.

### 4.2 Exploration: emotional and cognitive foundations

Exploration constitutes the bedrock of clients’ self-healing, as it establishes the relational foundation necessary for clients to engage in deeper therapeutic work (Hill, 2020). Specifically, following Hill’s (2020) framework, exploration involves nurturing clients’ self-expression via rapport-building. Within this framework, major exploration strategies include approval and reassurance, as well as restatement. As interpreted here, the former addresses the emotional foundation and the latter the cognitive.

#### 4.2.1 Approval and reassurance

The approval and reassurance strategy is defined as verbal interventions aimed at providing encouragement and reinforcement (Hill, 2020). By its very nature, this strategy calls for wholehearted emotional endorsement.

##### 4.2.1.1 Metadiscourse distribution

Figure 2 indicates that the approval and reassurance strategy features a highly focused metadiscourse distribution. Engagement markers overwhelmingly dominate, far exceeding the next highest categories, self-mentions and attitude markers, which in turn stand well above the other seven categories. Within these categories, code glosses are nearly imperceptible, indicating that this strategy rarely elaborates on propositional content. In addition, this strategy records the highest normalized frequency of boosters among all six strategies.

A closer look at illustrative examples reveals two recurrent co-occurrence patterns: engagement markers paired with attitude markers (Example 1), and engagement markers embedded within a fuller configuration of attitude markers, self-mentions, and boosters (Example 2).

Example 1: 非常理解 (attitude marker) 你的 (engagement marker)心情, 抱抱你 (engagement marker).

Translation: Deeply understand (attitude marker) your (engagement marker) feelings. Hug you (engagement marker).

In Example 1, “理解” [understand] functions as an attitude marker, while “抱抱” [hug] serves as an affective verb within the propositional content, both associated with the engagement markers. Together they perform relational acts of affirming empathy and offering comfort.

Example 2: 我 (self-mention) 相信 (attitude marker) 你 (engagement marker) 一定 (booster) 能够变为自己喜欢的样子, 以最真实的自己来生活学习.

Translation: I (self-mention) believe (attitude marker) you (engagement marker) will certainly (booster) become who you want to be, living and learning as your truest self.

Likewise, in Example 2, the self-mention “我” [I] pairs with the attitude marker “相信”[believe], which takes the engagement marker “你” [you] as its recipient, while the booster “一定”[certainly] reinforces the acknowledgement. This pattern conveys a supportive and encouraging stance that bridges the counselor’s personal conviction and a positive, confident vision for the client’s future.

In both examples, “你” [you] serves as the focal point around which the other markers cluster, reinforcing its role as emotional address rather than neutral reference. These two examples illustrate how the strategy’s characteristic categories (engagement markers, attitude markers, self-mentions, and boosters) coalesce in actual discourse.

##### 4.2.1.2 Metadiscourse functions

As the preceding section indicates, approval and reassurance addresses the affective base of exploration, where two concerns are especially salient: clients’ uncertainty about whether their experiences merit therapeutic attention, and their fear of being overwhelmed by painful emotion (Kleiven et al., 2020). The characteristic pattern of engagement markers, attitude markers, self-mentions, and boosters responds to the above concerns along two complementary lines: affirmation and safety.

As for affirmation, the co-occurrence of engagement markers, attitude markers, self-mentions, and boosters can be read as a linguistic expression of the stance associated with “unconditional positive regard” (Rogers, 1992, p. 829). Engagement markers enact attentiveness by explicitly addressing the client. Attitude markers, self-mentions, and boosters then imbue this attention with affective recognition, personal endorsement, and positive belief, together realizing a caring, affirmative stance. Together they establish an inclusive, non-judgmental atmosphere that remains available even when clients recount distressing or shameful experiences. In this way, the approval and reassurance process is presented as unconditional rather than contingent on the acceptability of what clients disclose. Such affirmation is oriented toward alleviating clients’ concerns about whether their experiences merit therapeutic attention. This is consistent with evidence that clients tend to evaluate therapists more favorably when they are perceived as warm, encouraging, and affirmative (Di Malta et al., 2025). Prior mixed-methods research similarly found that humanistic therapists use second-person pronouns and consistent acknowledgements of clients’ negative emotions to align with clients’ internal frames of reference (Qiu and Tay, 2023). The present research extends this line of work by showing how counselors apply engagement markers, attitude markers, self-mentions, and boosters within online counseling contexts.

As for safety, engagement markers and self-mentions jointly take on a function beyond what has been noted above: with self-mentions marking the counselor as the source of the utterance and engagement markers marking the client as its recipient, the pattern of “I” and “you” address casts the interaction as a private, mutually engaged exchange in which the client’s disclosure is met by an explicitly present listener. In doing so, it keeps the interaction within the immediate counselor-client relationship and constructs a “safe and bounded environment” (Lucherini Angeletti et al., 2025, p. 2), one in which painful emotions can be freely voiced by clients and securely held by counselors. This environment resonates with the relational qualities of “mutual awareness” (Liu and Cheng, 2025, p. 26) and “perceived helpfulness” (Huang et al., 2025, p. 2). This can be interpreted as creating a felt sense of safety in which clients may approach their true feelings without being overwhelmed, which could help furnish the emotional prerequisite for their opening up to self-exploration.

#### 4.2.2 Restatement

Restatement denotes counselors’ recasting of clients’ cognitive substance in their own wording, apart from its emotional undertones (Hill, 2020). In essence, this practice constitutes the co-description of clients’ subjective experiences proposed by Weiste et al. (2016), which allows clients to reflect on their cognitions from a rational standpoint to facilitate self-exploration.

##### 4.2.2.1 Metadiscourse distribution

Figure 3 reveals a dual-peak distribution: engagement markers and transitions form two prominent spikes, while the remaining categories recede into comparatively low frequencies. While this configuration shares a heavy reliance on engagement markers with the approval and reassurance strategy, restatement is distinguished by its comparatively high frequency of transitions.

A deeper probe beneath this dual-peak structure reveals that engagement markers and transitions frequently co-occur alongside clients’ already disclosed factual conditions. An illustrative instance is presented below:

Example 3: 然而 (transition) 这种压抑, 并没有促进事情的完成, 现实中的你 (engagement marker) 虽然 (transition) 觉得应该完成那些事, 最终却 (transition) 很少做到.

Translation: However (transition), this suppression did not actually help things get done; in reality, although (transition) feeling obligated to complete those tasks, you (engagement marker) still (transition) end up rarely doing so.

In Example 3, the engagement marker (“你” [you]) is tightly interwoven with propositional content rooted in the client’s own stated intentions and self-described struggles, positioning the client as directly addressed and personally involved. The transitions (“然而” [however], “虽然” [although], “却” [still]) complement this relational function via contrastive elaboration and likewise co-occur with the client’s pre-existing described experience: “虽然” [although] introduces the client’s felt obligation, whereas “却” [still] and “然而” [however] mark the discrepancy between what the client intends and what actually happens. Collectively, these transitions logically connect the client’s stated distress, restructuring a fragmented account of intention and inaction into a cohesive sequence sourced from the client’s original narration, reflecting restatement’s cognitive orientation.

##### 4.2.2.2 Metadiscourse functions

As noted above, restatement works with the cognitive substance of what clients have said, recasting it in the counselor’s words. The dual-peak profile identifies engagement markers and transitions as the strategy’s main metadiscourse resources. These observations point to two purposes: (1) faithfully mirroring clients’ expressions through client-centered engagement, and (2) structuring their content through logical reorganization. These purposes correspond to the functions realized by the strategy’s metadiscourse distribution: experiential mirroring and narrative structuring.

First, experiential mirroring is realized mainly through engagement markers, especially second-person references. These markers frequently emerge together with clients’ previously disclosed contents. The significance of this pattern lies in its focus: “you” directs the client’s attention back to the experience they have described, detaching it from the immediate flow of talk and holding it up for renewed reflection. In this way, it prompts the client to retrieve and confirm what they have said. This invites “a shared observational position” (Wahlström, 2023, p. 2), in which the counselor and the client immerse themselves in that experience as something to be revisited and understood. A further layer concerns the way second-person references put “the client more clearly ‘on stage’ as the target of the talk” (Etelämäki et al., 2021, p. 10), thereby making the client the explicit participant in the counselor’s utterance. This can be interpreted as an orientation toward making clients feel “heard, valued, and accepted” (Li, 2025, p. 145). This emphasis on making the client’s experience the object of reflection echoes the observation that “making a person’s own experience the focus of their empathic self-understanding is indeed the focus of psychotherapy” (Irarrázaval and Kalawski, 2022, p. 4). By virtue of these efforts, counselors endeavor to fully perceive and faithfully mirror clients’ experiences, an orientation consonant with clients’ self-exploration.

Second, narrative structuring is realized mainly through transitions. Clients’ thoughts often remain too fragmented to articulate, leaving their inner experiences difficult to express (Kleiven et al., 2020). Transitions address this fragmentation by linking separate pieces of the client’s account into an ordered sequence. This use of transitions parallels diagnostic reformulations, in which therapists reconstruct a clearer account of clients’ circumstances and clarify the nature of the problem at hand (Antaki et al., 2005). In the present corpus, transitions perform this work by showing how successive claims relate to one another, so that the client’s initially scattered account becomes a coherent line of reasoning. This coherence brings previously implicit connections into view. The pattern aligns with Knol et al. (2020), who observed therapists using discourse markers such as “so,” “but,” and “and” to reformulate intricate patient recounts into manageable units of talk. Thus, transitions in restatement function as a structuring device: by disentangling chaotic thoughts and marking the links between pivotal issues, they present clients’ underlying concerns in a clearer sequence, a step toward their self-exploration.

### 4.3 Insight: meaning-making through interpretation

Insight aims to assist clients in uncovering the root causes behind their behavioral patterns, moving them beyond surface-level awareness toward deeper self-understanding (Hill, 2020). Interpretation serves as one of the key insight strategies (Hill, 2020), contributing to meaning-making in the therapeutic process.

Interpretation offers alternative explanations and relational connections that extend beyond clients’ explicit verbal accounts (Hill, 2020). In other words, it seeks to “interpret the unconscious meanings of what clients have said” (Suomalainen et al., 2023, p. 53).

#### 4.3.1 Metadiscourse distribution

Figure 4 indicates that engagement markers and transitions constitute the two most frequent metadiscourse categories within interpretation. Moreover, the most notable feature is the unexpected presence of hedges in the top three, a category that is relatively infrequent in most other strategies but similarly elevated in direct guidance.

Further analysis of illustrative examples reveals that engagement markers, transitions, and hedges converge alongside counselors’ newly inferred interpretive content beyond clients’ pre-stated information, collectively structuring the counselors’ inferential reasoning. An illustrative case is provided below:

Example 4: 因为 (transition) 这些事情只是事情, 你的 (engagement marker) 认知可能 (hedge) 会认为这些事情是非常糟糕的, 然而 (transition) 过去的事情已经发生甚至 (transition) 并不是那么糟糕, 所以 (transition) 需要释放宣泄出来, 允许五分钟的打转觉察, 时间到了就要及时抽身.

Translation: Because (transition) these things are just things, your (engagement marker) cognition may (hedge) perceive them as terribly disastrous; however (transition), what has already happened is not even (transition) that bad, so (transition) you need to release and vent it, allowing yourself 5 min of dwelling and awareness, then promptly step away once the time is up.

In Example 4, the three metadiscourse categories work in concert. The engagement marker (“你的” [your]) ties the counselor’s reframing directly to the client’s cognitive appraisal, inviting the client to reconsider how their cognition may have been shaping a catastrophized reading of past events. The transitions (“因为” [because], “然而” [however], “甚至” [even], and “所以” [so]) then link the client’s presumed appraisal, the counselor’s alternative reading, and the resulting suggestion into an organized and understandable reasoning chain, assembling the counselor’s reinterpretation step by step. Notably, the hedge (“可能” [may]) marks the counselor’s characterization of the client’s cognition as a provisional conjecture rather than a factual assertion about the client’s inner state. By framing this cognitive appraisal as an open-ended possibility subject to the client’s revision, the hedge renders what could otherwise be considered one-sided expert correction as collaborative meaning-making, respecting client subjectivity and encouraging the client to participate in reconsidering the negative appraisal.

#### 4.3.2 Metadiscourse functions

Interpretation, as defined above, offers alternative explanations and relational connections that extend beyond clients’ explicit verbal accounts. Its distribution profile highlights three dominant resources: engagement markers, transitions, and hedges. These definitional and distributional cues converge on three purposes: (1) guiding clients toward perspectives they have not yet recognized, (2) clarifying latent connections, and (3) softening explanatory claims through tentative wording. The discussion below examines these purposes as interpretive guiding, connective clarifying, and epistemic softening.

First, interpretive guiding is realized mainly through engagement markers, especially second-person pronouns. These markers attach the counselor’s newly developed insights directly to the client, linking an explanation that extends beyond the client’s stated content to the client’s own experience. This direct address turns a potentially abstract interpretation into a personally relevant reflection and invites the client to consider factors beyond their original cognitive scope. Such participation echoes the notion of second-person relatedness, which holds that sustained second-person interaction supports mutual recognition and joint reflection (Vanney and Sáenz, 2021). In therapeutic dialogue, this logic suggests that second-person address opens room for shared meaning-making in which the client takes part in the emerging interpretation.

Second, connective clarifying is realized mainly through transitions, which arrange the counselor’s analytical observations into an ordered chain of reasoning. Through this ordering, they make explicit how different pieces of the counselor’s interpretive account fit together, including connections that had remained outside the client’s awareness. This use of transitions matches the view that discourse connectives act as processing instructions, easing the reader’s need to infer coherence relations (Crible and Pickering, 2020). In the context of insight, this arrangement gives clients a clearer basis for arriving at new understandings on their own terms.

Third, epistemic softening is realized mainly through hedges, which present the counselor’s explanation as provisional. By holding back excessive inference and overstatement, hedges create space between the counselor’s perspective and the client’s judgment, leaving room for the client to weigh the interpretation for themselves. In this sense, interpretation can be positioned as an intersubjective process. Such a view is compatible with Del Giacco et al.’s (2020, p. 21) caution against counselors’ “affirming with conviction a particular state of reality”, as overly assertive statements risk relational ruptures and client resistance. Rather, counselors may benefit from adopting a decentered stance by acknowledging clients’ “primary authorship status” (Rhodes, 2020, p. 83) instead of imposing authoritative perceptions. This use of hedges also accords with the principle of tentativity identified by Stukenbrock et al. (2021) that therapists tend to present interpretations as open and negotiable rather than definitive. Together, these accounts suggest that interpretation is most coherent with a tentative, respectful approach that leaves the client room to pursue their own process of understanding.

### 4.4 Action: lowering the threshold for change

Action supports clients in moving toward concrete behavioral change. Counselors guide clients to pursue change and collaboratively develop actionable plans for behavioral adjustment (Hill, 2020). The major strategies converging to serve this function include information and direct guidance (Hill, 2020), with the aim of lowering the threshold for change.

#### 4.4.1 Information

Information supplies clients with essential knowledge and factual input (Hill, 2020), equipping them with the context necessary to understand their situation and take purposeful action toward change.

##### 4.4.1.1 Metadiscourse distribution

Figure 5 presents a notable reversal: transitions displace engagement markers from the top position. The top three items are transitions, engagement markers, and frame markers. Meanwhile, the lower overall frequency profile aligns closely with the nature of the information strategy, which centers on delivering propositional content. Self-mentions are markedly lower in this strategy than in any other, consistent with the strategy’s orientation toward objective content rather than the counselor’s own perspective. In contrast, evidentials are comparatively more frequent here than in most other strategies, reflecting their function of grounding information in external sources of knowledge.

In the information strategy, the three most prevalent categories of metadiscourse regularly appear together with factual content to construct structured knowledge delivery. Together, these metadiscourse resources shape raw information into well-organized explanations, illustrated below through the convergence of the top three categories (Example 5) and, more specifically, through the strategy’s characteristic engagement pattern (Example 6):

Example 5: 关于 (frame marker) 遗忘的原因, 比较主流的有四种说法, 分别是: 1, (frame marker) 记忆痕迹衰退说也叫自然衰退说 2, (frame marker) 干扰抑制说 3, (frame marker) 线索依赖型遗忘 4, (frame marker) 动机性遗忘又叫选择性遗忘, 这个和你 (engagement marker) 说的“情绪影响记忆”有些相似, 但 (transition) 又不完全相同.

Translation: As for (frame marker) the causes of forgetting, there are four mainstream explanations: 1, (frame marker) the trace decay theory, also called natural decay theory; 2, (frame marker) interference theory; 3, (frame marker) cue-dependent forgetting; 4, (frame marker) motivated forgetting, also called selective forgetting. This is somewhat similar to what you (engagement marker) said about “emotions affecting memory,” but (transition) not entirely the same.

In Example 5, the frame markers (e.g., “关于” [as for] and the enumerating numerals “1,” “2,” “3,” “4”) organize the causes of forgetting into four clearly delineated units, rendering the breakdown of theoretical explanations explicit and structurally accessible. The engagement marker (“你” [you]) then links this professional account back to the client’s own prior description, framing the delivered knowledge as directly relevant to, rather than separate from, the client’s existing understanding. In turn, the transition (“但” [but]) qualifies this link, distinguishing the client’s intuitive account from the professional classification without dismissing it outright. Collectively, these markers deliver factual content as structured, accessible knowledge while maintaining relevance to the client’s experience.

A closer examination of the lexical composition of engagement markers reveals that the inclusive “我们” [we] outnumbers the second-person “你” [you] in this strategy, the only one of the six strategies in which this ordering occurs. In particular, “我们” [we] functions as an inclusive pronoun that folds the client and the counselor into a shared group, as illustrated in the following example:

Example 6: 我们 (engagement marker) 能够理解他人情感时运用的就是我们的 (engagement marker) 同理心, 也就是 (code gloss) 共情, 而 (transition) 这些情感也都是发自内心真实的感受, 共情让我们 (engagement marker) 与他人建立起 “情感”联系.

Translation: What we (engagement marker) use to understand others’ emotions is precisely our (engagement marker) empathic capacity, that is (code gloss), empathy; and (transition) these emotions are also genuinely felt from the heart, and empathy lets us (engagement marker) build “emotional” connections with others.

In Example 6, the engagement marker “我们” [we], recurring three times, folds the client into a shared group that possesses this capacity, implicitly inviting the client to recognize it as their own. Here, “我们” [we] refers to the writer and the audience, namely the counselor and the client, not the broader helping profession, and is therefore coded as an engagement marker, not a self-mention. If the counselor had used a generic term like “人们” [people], the same claim would still hold, but the client would remain an external observer rather than a participant. “我们” [we] accomplishes both: it states the proposition while also positioning the client within it. In addition, the code gloss “也就是” [that is] then substitutes the more precise term “共情” [empathy] for the preceding, more general “同理心” [empathic capacity], recasting the counselor’s initial wording into the professional term. The transition “而” [and] subsequently extends this account, connecting the claim that such feelings are genuinely felt to the further claim that empathy builds “emotional” connections with others, broadening the explanation from an internal capacity to its interpersonal consequence. Taken together, these markers perform the depersonalizing work that sustains consensus building. The client’s emotional responsiveness is repositioned as an instance of a universally shared psychological phenomenon rather than an individual idiosyncrasy.

These two examples illustrate the two engagement patterns characteristic of this strategy: personalization through the second-person “你” [you], and consensus building through the inclusive “我们” [we]. Both pair with transitions, mirroring the frequency profile shown in Figure 5.

##### 4.4.1.2 Metadiscourse functions

As introduced earlier, information delivery supplies established, digestible knowledge that clients need before they can act, and the strategy’s high-frequency metadiscourse markers echo this characteristic. Three action-related needs shape the strategy: (1) when clients struggle to assemble separate facts into a clear view of their situation, transitions provide logical linking; (2) when they are unsure what to do, engagement markers build consensus by presenting guidance as shared professional knowledge; and (3) when the information load exceeds the threshold of what clients can readily process, frame markers break the content into smaller, more manageable units for informational elaboration.

First, logical linking is realized mainly through transitions. These markers make the logical structure of the information visible by signaling how successive facts, viewpoints, and options fit together, so that the counselor’s account unfolds in a clear, layered sequence. This use of transitions is consonant with psycholinguistic evidence that readers draw on connectives to recognize the coherence relations that hold a text together (Scholman et al., 2024). In counseling contexts, transitions perform a comparable organizing role: by marking the links between the individual points being delivered, counselors give clients a basis for assembling them into a coherent understanding of their situation. A clearly ordered account also brings the logical progression into view, so clients can follow the counselor’s reasoning without having to supply missing connections. This visible path links understanding more directly to the concrete behavioral steps that follow.

Second, consensus building is realized mainly through engagement markers. These markers show a stronger preference for the inclusive we than they do in the other strategies. The inclusive we recasts what might otherwise appear as an individual counselor’s opinion into shared, commonly endorsed knowledge. This shared understanding parallels a stage in the Common-Sense Model of Self-Regulation (McAndrew et al., 2018), which holds that therapists and clients need to reach a common understanding of the client’s condition before deciding on a course of action. For action, this consensus-building is especially relevant. When a difficulty is presented as a common psychological experience, the recommendation may no longer appear as a judgment of the individual. Clients can then turn their attention to what they might do, without first spending emotional energy questioning their adequacy. This shared account further separates the process of absorbing information from the fear of being blamed. With that fear lessened, clients can focus on understanding and evaluating the advice, making the step from receiving information to considering action more direct.

Third, informational elaboration is realized mainly through frame markers. These markers introduce, delimit, or conclude topics, defining the scope of the professional knowledge being presented and marking the boundaries of an ongoing explanation. By partitioning complex explanations into numbered or sequenced units, they divide dense information into smaller segments and make the structure of the delivery easier to follow. This step-by-step presentation is broadly consistent with calls to treat informed consent as a procedural, ongoing process, beyond a single disclosure event (Blease and Kelley, 2018). Health behavior change research has likewise associated structured segmentation with improved engagement and performance when working memory would be exceeded (Baxter et al., 2025). Although these findings come from health behavior contexts, the underlying mechanism is analogous: frame markers perform a comparable chunking role, breaking overwhelming content into discrete units that clients can process one at a time. By elaborating dense content into separate segments, frame markers make the information more manageable, so that clients can examine each segment sequentially before using it in decisions about behavioral change.

#### 4.4.2 Direct guidance

Direct guidance offers clients concrete suggestions regarding their subsequent coping and behavioral steps (Hill, 2020). In practice, it aims to provide clients with advice and guidance that they can act on.

##### 4.4.2.1 Metadiscourse distribution

Direct guidance’s metadiscourse distribution is led by engagement markers, followed by transitions, showing a client-directed, well-structured mode of advice-giving. What stands out, however, is that hedges, rather than frame markers, complete the top three, echoing interpretation.

Further observation reveals that in the direct guidance strategy, these three top-ranked metadiscourse resources often co-occur with concrete behavioral advice to build practical guidance, as seen in the following example:

Example 7: 所以 (transition) 你 (engagement marker) 可以 (hedge) 试着先调整一下自己的状态, 看看自己的情绪是否稳定, 自己的睡眠是否正常.

Translation: So (transition) you (engagement marker) could (hedge) try first adjusting your own state, and check whether your emotions are stable and your sleep is normal.

In Example 7, the transition “所以” [so] ties this piece of advice back to the preceding diagnostic discussion, presenting the suggestion as a logical consequence rather than an arbitrary directive. The engagement marker “你” [you] then positions the client as the direct recipient of this guidance. The hedge (“可以” [could]) softens the command-like tone, turning it into an open invitation, leaving room for the client to decide whether, and how, to act on it. This contrasts with a stronger modal such as “必须” [must], which would frame the action as obligatory.

##### 4.4.2.2 Metadiscourse functions

As outlined earlier, direct guidance offers clients concrete suggestions regarding subsequent coping and behavioral steps, and its metadiscourse distribution is led by engagement markers, transitions, and hedges. To advance action, direct guidance is (1) tailored to the client’s specific circumstances, (2) arranged into a coherent action plan, and (3) qualified to preserve the client’s latitude in accepting it. These communicative requirements give rise to three features: targeted personalization, procedural coherence, and directive tempering.

First, targeted personalization is realized mainly through engagement markers. These markers present direct guidance as tailored to the individual client, turning potentially impersonal advice into a consultative dialogue attuned to the client’s situation, since clients tend to prefer personalized therapy to a uniform approach (Li E. et al., 2025). This personalization is especially pertinent to action: when the advice appears designed for the client’s situation, the behavioral steps may seem more applicable, and clients may be more inclined to follow through. As Many et al. (2024, p. 166) observe, “personalized terms engage patients more effectively, fostering a sense of involvement and empowerment in their healthcare decisions. The role of engagement markers here differs from that in information, the other action-oriented strategy. In information, they build a shared understanding that prepares the ground for subsequent directives; in direct guidance, they present the suggested action as something the client can personally commit to.

Second, procedural coherence is realized mainly through transitions. These markers arrange sequential steps, alternative plans, or operational procedures into a coherent order, so that separate directives form a single line of action. This ordering can make the progression from one step to the next easier to follow: when clients can see how each instruction leads to the next, they may be better able to understand and act on the sequence free from confusion or overload. Psycholinguistic research offers a comparable account: discourse connectives serve as procedural instructions that signal how adjacent stretches of discourse are related, enabling readers to process congruent sequences more readily than incoherent ones (Wetzel et al., 2022). In other words, the presence of explicit connectives can lighten the processing burden of inferring relational structure, leaving more cognitive capacity for the content itself. The underlying principle transfers: transitions in direct guidance mark the logical order in a comparable way, making it more perceptible to clients.

Third, directive tempering is realized mainly through hedges. These markers soften the illocutionary force of the advice itself, so that advice that might be experienced as a command is offered as an option the client is free to take up, adapt, or decline. This role differs from the epistemic softening observed in interpretation: there, hedges moderate the extent to which a novel explanation is asserted; here, they moderate the strength of obligation conveyed when a course of action is presented, shifting the utterance from directive to suggestive. Consistent with this, Akıncı Oktay (2020) found that hedges in advice-giving discourse soften the force behind the advice itself. Two explanations have been proposed for this pattern: hedging shields the advice-giver, since a given piece of advice may admit multiple interpretations, and it protects the recipient’s face by leaving room for the suggestion to be accepted or declined. For action, the value of tempering lies in the option it preserves: when behavioral suggestions appear as available options, not binding obligations, clients may be more inclined to engage with them in light of their situation, which may make the suggested steps easier to take up than to resist or ignore.

### 4.5 Across exploration, insight, and action: resonant narratives through self-disclosure

Exploration, insight, and action each involve different variants of counselors’ self-disclosure. It is a strategy through which counselors “use their self in therapy” (Alva et al., 2025, p. 22). This strategy encompasses several subtypes: disclosure of feelings, disclosure of similarities, disclosure of insight, and disclosure of strategies (Hill, 2020). All these subtypes share a common trait: they entail references to the counselors themselves. Proper self-disclosure proves instrumental in nurturing the therapeutic relationship and consolidating the therapeutic alliance (Barnett, 2011).

#### 4.5.1 Metadiscourse distribution

Self-disclosure in the PsyQA dataset (Sun et al., 2021) is annotated as a single category. Figure 7 therefore presents the aggregated metadiscourse distribution of self-disclosure as a whole. Self-mentions tower over all other categories, a dominance that follows in part from Hill’s (2020) definition of self-disclosure as counselors’ references to themselves. More revealing is the second-ranked category: transitions, which considerably outpace engagement markers despite the latter dominating most other strategies. This pattern suggests that self-disclosure functions less through direct address to the client and more through narrative logic, as counselors construct their personal accounts. The following example illustrates how self-mentions and transitions jointly unfold in practice:

Example 8: 我 (self-mention) 现在对自己依然要求高, 但是 (transition), 我 (self-mention) 有点发现, 我 (self-mention) 不必每时每刻都优秀, 都在做无解的事, 我 (self-mention) 可以留点放松的时间, 这样对大脑的过量使用, 是有助于恢复的, 反而 (transition) 效率更高.

Translation: I (self-mention) still hold myself to high standards, but (transition) I (self-mention) have come to realize that I (self-mention) do not have to be excellent every moment or keep taking on tasks with no clear solution. I (self-mention) can leave myself some time to relax; doing so helps the brain recover from overuse, and instead (transition) I end up more efficient.

In Example 8, two transitions mark successive turns in the counselor’s reasoning: “但是” [but] introduces a realization that interrupts an initial stance of high self-expectation, and “反而” [instead] reverses the expected relationship between rest and productivity. Self-mentions, appearing four times, keep this reasoning rooted in the counselor’s first-person reflection. Such reliance on self-referential, transition-marked reasoning, with less direct address to the client, is the feature that most clearly sets self-disclosure’s discursive profile apart from the other strategies.

Based on a close reading of the corpus, we found that transitions perform a comparatively stable role: they organize counselors’ self-disclosure narratives into a coherent sequence of reasoning, linking successive turns in the counselor’s reflection or life account, as illustrated in Example 8. Self-mentions, by contrast, show more variation; the following section examines how their roles shift among the exploration, insight, and action subtypes.

#### 4.5.2 Metadiscourse functions

Overall, self-mentions form the common linguistic foundation of self-disclosure, marking counselors’ personal revelations as part of the discourse. Their discursive role, however, changes with the content disclosed.

First, self-disclosure of feelings and similarities serves exploration by conveying peer affiliation. These subtypes draw on counselors’ affective experiences and parallel life circumstances to offer what Schriver et al. (2022, p. 45) call “my side tellings,” which bring the counselor’s perspective closer to the client’s experience. Self-mentions thereby create a peer-like presence, hinting that even trained professionals have faced comparable challenges. This presence may soften clients’ isolation and create a sense of belonging that is conducive to voluntary self-exploration. Moreover, such disclosure presents the counselor less as a distant authority and more as a fellow human who has faced similar experiences. This presentation may encourage perspective-taking and cultivate a “reciprocal relationship” (Lichtenstein, 2025, p. 293) in which both participants openly exchange subjective experiences. This reciprocity is consistent with computational evidence that greater semantic alignment between client and therapist, alongside richer conversational dynamics, characterizes higher-quality engagement in therapeutic dialogue (Rueda et al., 2025).

Second, self-disclosure of insight serves insight by indirect illumination. This subtype involves counselors sharing how their thinking changed and guiding clients toward fresh understandings (Hill, 2020). Clients may develop insight more readily when they can observe another individual working through confusion toward clarity. Without offering explicit interpretations, counselors draw on personal experiences to open a possible route toward understanding that clients can follow. Self-mentions realize this process linguistically: they convey the counselor’s past confusions, struggles, and the specific ways clarity was eventually achieved. Through this disclosure, self-mentions can make the counselor’s vulnerability and authenticity perceptible, which may inspire clients to gain insight into their situations. These self-mentions may generate empathic resonance and create a safer, more trust-laden therapeutic atmosphere.

Third, self-disclosure of strategies serves action by demonstrating feasibility. This subtype refers to counselors sharing past coping strategies for clients’ reference (Hill, 2020). For this disclosure to prompt action, self-mentions provide the main metadiscourse resource through which the suggested strategies become visible as practically workable. By presenting themselves as living evidence that difficulties can be navigated and changes achieved, counselors use their lived experiences to “model for clients” (Rachwal and Gredecki, 2026, p. 39), which may make behavioral change feel more attainable. Moreover, when clients recognize that even trained practitioners have relied on deliberate strategies to make progress, their sense of helplessness may lessen and their belief in their capacity for progress may strengthen. Self-mentions may “normalize coping strategies” (Hume, 2022, p. 157): they can convey interpersonal affinity and make feasible, sustainable behavioral shifts seem more accessible.

Of note, the high frequency of self-mentions observed in our research seems to diverge from findings in spoken psychotherapeutic contexts. For instance, Ryu et al. (2023) found that therapists’ frequent use of first-person pronouns in such settings correlated negatively with patient-rated therapeutic alliance. Because therapeutic alliance was not measured in this research, we offer a possible contextual explanation for this divergence. In a supportive, client-centered online environment, self-mentions may act as a moderate form of self-disclosure instead of self-preoccupation, and can serve as a way of building rapport through shared experiences. This interpretation is consistent with Li M. et al. (2025), who found that support providers’ first-person language, often used when they draw on similar experiences, positively predicts cognitive change among help-seekers in online mental health communities, an effect the authors attribute in part to the sense of perceived similarity and emotional empathy it fosters. Taken together, these findings raise the possibility that, in online contexts, self-mentions may serve as a rapport-building resource, an interpretation that future research could test by measuring alliance in this context.

## 5 Conclusion

### 5.1 Contributions

Empirically, this research represents an attempt to provide evidence on counselors’ metadiscourse use in the underexplored field of Chinese text-based online counseling. It also offers a preliminary step toward bridging the research strands of metadiscourse analysis and counseling strategy research. First, it maps metadiscourse distributions for each counseling strategy, suggesting that each strategy relies on an identifiable, function-driven linguistic configuration rather than arbitrary language selection (addressing RQ1). Second, it elaborates how these metadiscourse distributions function to support exploration, insight, and action (addressing RQ2). By bringing these two fields into dialogue, this research considers counselors’ language use as a structured, function-driven system aligned with the goals of each counseling strategy.

Theoretically, this research contributes to the expanding literature on psychotherapy and discourse analysis through two layers: (1) Uncovering the linguistic realization of Hill’s (2009, 2020) Helping Skills. This research supplements the framework by bringing to light that therapeutic intentions are associated with specific metadiscourse distributions, offering a language-based lens to understand how helping skills unfold in text-only environments. (2) Applying Hyland’s (2005) Interpersonal Model of Metadiscourse and suggesting a possible extension. This research illustrates the applicability of the interpersonal metadiscourse framework to an affect-intensive, relationship-oriented psychological context. Our findings further suggest that the same metadiscourse category may undertake different, context-specific functions depending on the counseling function it serves. For instance, engagement markers shift from emotional address in approval and reassurance to targeted personalization in direct guidance, while self-mentions serve peer affiliation, indirect illumination, and feasibility demonstration across the subtypes of self-disclosure. These functional differences constitute the substantive contribution of the present analysis.

### 5.2 Implications

#### 5.2.1 Theoretical implications

This research suggests that online psychological counseling is a discursively constructed event. The use of metadiscourse may shape how advice is received. This suggests that the linguistic form of counseling responses is worth examining alongside their propositional content.

#### 5.2.2 Practical implications

Our findings identify metadiscourse patterns associated with different counseling strategies, which may serve as practical references for novice counselors. In addition, generative models could potentially be trained to reproduce these descriptively observed metadiscourse configurations; whether these outputs are also therapeutically effective remains to be empirically tested.

### 5.3 Limitations and future research

First, this study draws on the PsyQA dataset sourced from a single online counseling platform. Though the counselors’ responses are ecologically valid, platform-specific discursive norms may shape linguistic practices to some degree. Future research could incorporate corpora from multiple platforms to verify the generalizability of the present findings. Second, the analysis focuses primarily on lexical-level metadiscourse markers. While effective for identifying interpersonal features, this approach leaves syntactic structures and paralinguistic cues (e.g., emojis, expressive punctuation) for future work. Third, this research adopts a descriptive rather than predictive design. Because therapeutic alliance and client outcomes were not directly measured, the present data are insufficient to demonstrate that these functional interpretations translate into actual therapeutic effect. Future work could further explore the relationship between metadiscourse and therapeutic outcomes.

## Data Availability

The datasets used in this study are not currently available publicly. More information can be found at: https://github.com/thu-coai/PsyQA. Further inquiries can be directed to the corresponding author.
